# Increasing Utilization of Quadriceps Tendon Autograft in Adult Primary Anterior Cruciate Ligament Reconstruction: A Retrospective Review

**DOI:** 10.7759/cureus.87352

**Published:** 2025-07-05

**Authors:** Timothy Foster, Brandon Klein, Shebin M Tharakan, Lucas E Bartlett, Aaron Atlas, Randy Cohn, Nicholas A Sgaglione

**Affiliations:** 1 Orthopaedic Surgery, Northwell Health, New Hyde Park, USA; 2 Orthopaedic Surgery, New York Institute of Technology College of Osteopathic Medicine, Oyster Bay, USA

**Keywords:** anterior cruciate ligament injuries, anterior cruciate ligament reconstruction, autografts, knee joint, quadriceps tendon

## Abstract

Introduction

Recent literature has demonstrated that the use of quadriceps tendon (QT) autograft for anterior cruciate ligament reconstruction (ACLR) is a viable alternative to more well-established autograft options. This study evaluated trends in QT autograft utilization in primary ACLR at a single institution.

Methods

A retrospective review of 2,352 ACLRs from 2014 to 2022 identified 1,582 primary ACLRs using autograft, after excluding allograft (651) and revision cases (119). The cohort included 1,021 men (66%) and 526 women (34%) with an average age of 24.0 years (range, 9-66). Time series analyses assessed annual quadriceps tendon utilization, and Pearson correlations identified trends, with significance defined as p<0.05.

Results

QT autograft was utilized in 3.7% (58/1,582) of autograft ACLR procedures and was used at least once by 29.7% (22/74) of surgeons. QT autograft was not utilized prior to 2020. Beginning in 2020, both the annual proportion of cases that utilized QT and the annual proportion of surgeons who utilized QT increased each year among all surgeon cohorts. QT utilization and hamstring tendon (HT) autograft utilization were inversely correlated among overall surgeons (R=-0.999, p=0.02) and among non-sports-trained surgeons (R=-0.999, p<0.01). HT autograft utilization decreased after the adoption of QT autograft at the study institution among sports-trained surgeons (R=-0.998, p=0.037).

Conclusion

Quadriceps tendon utilization for primary anterior cruciate ligament reconstruction has increased at a single institution since 2020 and corresponded with declining hamstring tendon use across surgeon cohorts with varying training, experience, and case volumes.

## Introduction

Graft selection remains a major consideration in the preoperative planning of anterior cruciate ligament reconstruction (ACLR), with operative decisions dependent on patient age, gender, level of activity, and surgeon preference [1,2]. Trends in graft utilization have been driven by functional outcomes, postoperative morbidity, and an improving understanding of the structure and biomechanics of the native ACL [3-5]. Historically, autograft ACLR has largely relied on the utilization of the bone-patellar tendon-bone (BPTB) or the hamstring tendon (HT). However, recent literature has demonstrated that the quadriceps tendon (QT) autograft is a viable, alternative to these well-established options with several potential clinical benefits [3,6,7].

The BPTB has long been considered the “gold standard” for graft selection in autograft ACLR due to excellent rates of return to pre-injury activity level [8]. However, the BPTB autograft may be associated with higher rates of donor-site morbidity when compared to other graft choices [6,7,9,10]. Utilization of the HT autograft has been demonstrated to decrease donor-site morbidity and kneeling pain when compared to the BPTB, but HT graft diameter is unpredictable, which may contribute to higher rates of graft failure [11,12]. Further, some studies have reported higher rates of infection, weakness in terminal knee flexion, and graft laxity with the use of HT autograft [11-15]. Due to these concerns, the QT autograft has gained recent popularity as an alternative soft tissue graft choice. After Fulkerson and Langeland's technical note in 1995 detailed the successful use of a full-thickness QT autograft for ACLR, many biomechanical studies have further supported that the QT is a viable alternative [3-5,16,17]. The QT autograft has been shown to provide similar or improved functional outcomes to HT autografts for ACLR, with comparable reductions in donor site morbidity from the BPTB autograft [6,18-21].

Despite literature supporting the utilization of the QT autograft, its incorporation into clinical practice has been limited [22-24]. Therefore, we conducted a retrospective study at a single large, multi-center institution to evaluate autograft selection patterns in ACLR. The primary objective was to assess trends in QT autograft utilization from 2014 to 2022, focusing on its increasing adoption since it was first introduced into clinical practice at the study institution in 2020. The secondary objective was to identify surgeon characteristics associated with QT autograft adoption, including fellowship training, annual procedure volume, and years of experience. Trends were defined as changes in the proportion of ACLR cases using QT autograft annually, and surgeon factors were assessed via analyses that were stratified by fellowship training, procedure volume, and years of experience. Authors hypothesized that there would be increasing QT autograft utilization over the study period, which coincides with growing literature supporting its use.

## Materials and methods

After receiving institutional review board approval (#22-0551) from Northwell Health, New Hyde Park, USA, patients who underwent ACLR between January 1, 2014 and July 31 2022, were identified from a multi-centered institutional database using Current Procedural Terminology (CPT) code 29888 (arthroscopically aided anterior cruciate ligament repair/augmentation or reconstruction). Data were extracted manually from the electronic health record (EHR) by trained study personnel using a standardized data abstraction protocol. Database entries were independently reviewed by a second author to ensure accuracy. Patients who underwent ACLR using allograft and revision ACLR were excluded. Age, multiligamentous injury, or any specific comorbidity were not used as exclusion criteria.

**Figure 1:**
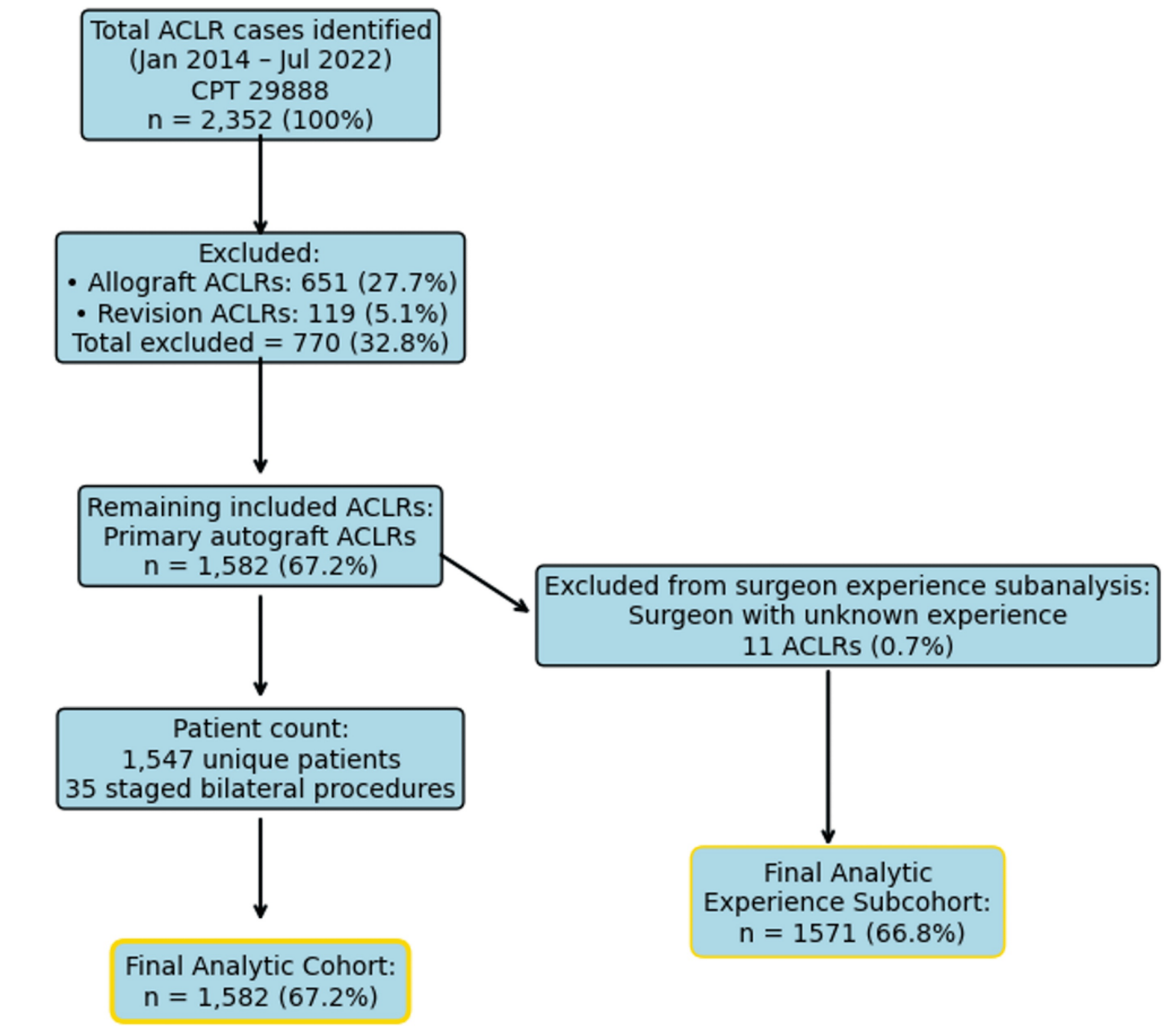
Flowchart of patient selection criteria and subgroup stratification for final analytic and experience-based cohorts. ACLR: anterior cruciate ligament reconstruction; CPT: Current Procedural Terminology, APV: annual procedure volume. Final analytic cohort includes all eligible primary autograft ACLRs. Surgeon experience subcohort excludes cases where annual volume was unknown.

Graft type (autograft versus allograft) and selection (BPTB, HT, quadriceps tendon (QT)) were recorded through thorough review of operative notes. Hybrid HT grafts (HT autograft reinforced with allograft tissue) were categorized as HT autografts since graft harvest was performed. Seventy-four surgeons with current New York State licensure were stratified into cohorts by fellowship training, annual procedure volume (APV), and years of clinical experience. Online institutional profiles and the New York State Physician webpage (https://www.nydoctorprofile.com/#search) were utilized to categorize surgeons based on fellowship training and years in clinical practice. Surgeons were categorized as sports-trained or non-sports-trained based on whether they had completed the Accreditation Council for Graduate Medical Education (ACGME) sports medicine fellowship. Surgeons were stratified by years in clinical practice as high-experience (15 years or more) or low-experience (fewer than 15 years), based on their number of years in clinical practice at the study’s midpoint (2018). This dichotomy utilized 15 years as the dividing point allowed for a comparable number of cases between cohorts. Surgeons were categorized into higher-volume (minimum 17 procedures per year) and lower-volume (less than 17 procedures per year) based on their APV of ACLR. APV was calculated by dividing the total number of ACLRs performed by the number of years that the surgeon performed at least one ACLR. Surgeon APV was dichotomized using a cutoff of 17 procedures per year based on recent demonstration of this volume as clinically significant by Schairer et al [25]. There were no missing data for primary variables of interest. Experience was unable to be determined for one surgeon (11 cases), which accounted for 0.7% of the total ACLR cohort. These cases were excluded only from analyses related to surgeon experience. No imputation was performed.

To assess changes in autograft selection before and after the first adoption of the QT, ACLR procedures were subcategorized by date of operation into pre-QT adoption (2014 to 2019) and post-QT adoption (2020 to 2022) cohorts. Statistical analyses were performed using Python 3.11.4 (Centrum voor Wiskunde en Informatica Amsterdam, The Netherlands) and Jamovi 2.4.6. (The jamovi project, Sydney, Australia). Descriptive characteristics were described as means with standard deviations. Categorical variables were expressed as the number of procedures, with proportions where appropriate. z-test of proportions was used to compare autograft utilization before and after 2020. Time series analyses were performed using Pearson correlation to assess trends in the annual proportion of autograft ACLR utilizing each autograft type. Comparative trend analysis was used to assess relationships between these trends. Statistical significance was defined as a p-value less than 0.05. Post hoc power analyses were conducted for the primary statistical tests (Pearson’s correlation and z-test for two proportions) using observed effect sizes, sample sizes, and α=0.05. Figures were created using Python 3.11.4 (Centrum voor Wiskunde en Informatica (CWI), Amsterdam).

## Results

Prior to the exclusion of 651 (27.7%) allograft ACLRs and 119 (5.1%) revision ACLRs, 2,352 total ACLRs were identified. Our remaining cohort of 1,582 ACLRs was performed on 1,547 patients (35 staged bilateral). The cohort ACLR was performed on 1,021 male patients (66.0%) and 526 female patients (34.0%), with an average patient age of 24.0 ± 8.4 years. Autograft ACLR was performed by 74 different orthopedic surgeons at the study institution. Of the surgeons, 48/74 (64.9%) were categorized as sports-trained, 37/73 (50.7%) as high-experience, and 71/74 (95.9%) as lower-volume. Non-sports-trained surgeons were trained in adult reconstruction (seven), pediatrics (five), trauma (one), hand (one), or did not complete a fellowship (12). The average surgeon experience was 14.3 ± 9.1 years (range: 2-36), and the average surgeon APV was 3.1 ± 4.6 ACLR per year (range: 1.0-28.7). Sports-trained surgeons performed 72.4% of the autograft ACLR, low-experience surgeons performed 57.5% of the autograft ACLR, and lower-volume surgeons performed 63.0% of the autograft ACLR.

**Figure 2:**
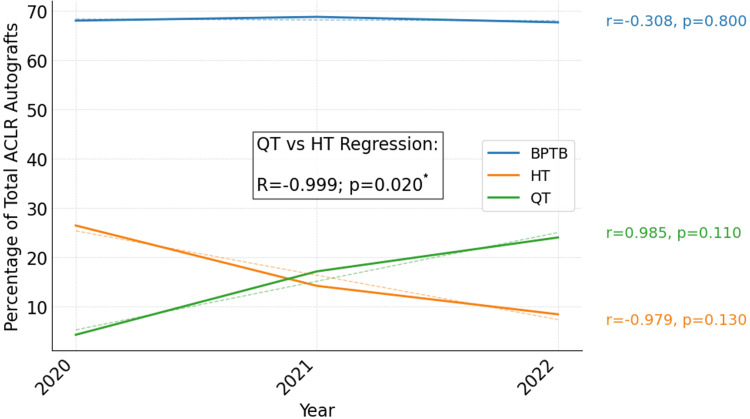
QT autograft utilization and HT autograft utilization were inversely correlated among all surgeons overall after QT adoption in 2020. * Indicates statistical significance using p<0.05 as significance threshold. R and p reflect results of Pearson correlation. Post hoc power analysis indicated the study had 99% power to detect this effect at α=0.05. ACLR: anterior cruciate ligament reconstruction, BPTB: bone-patellar tendon-bone, HT: hamstring tendon, QT: quadriceps tendon. Data has been represented as % (N/Annual total).

Over the entire study period, QT accounted for 3.7% (58/1582) of autograft ACLR and was utilized at least once by 29.7% (22/74) of surgeons. Only one QT autograft included a bone block, while all others were reported to be all soft-tissue grafts. There were no cases of autograft ACLR that utilized QT prior to 2020. There were subsequent increases in the annual proportion of autograft ACLR using QT, and the annual proportion of surgeons who utilized QT for autograft ACLR, after 2020 (Table 1). In 2021, 23.1% of operating surgeons (nine of 39) used QT autograft in the majority of their ACLR autograft procedures, which further increased to 25.8% (eight of 31) in 2022. Following the incorporation of QT autograft utilization, the proportion of cases using HT autograft decreased to 17.2% (70/408) from 40.3% (463/1150) before 2020 (p<0.01; z=-8.45) with moderate effect size (Cohen's h=-0.520). Over the period in which QT was utilized (2020-2022), the annual proportion of cases performed with QT showed an inverse correlation with HT utilization (R=-0.999, p=0.02) (Figure 2). HT utilization decreased significantly over the study period (R=-0.668, p=0.049). No trends were observed in the utilization of BPTB related to QT.

**Table 1:** Annual utilization of ACLR autografts (2014-2022). * Indicates statistical significance using p<0.05 as significance threshold. R and p represent statistical significance based on Pearson correlation (2014-2019 excluded for QT). ACLR: anterior cruciate ligament reconstruction, BPTB: bone-patellar tendon-bone, HT: hamstring tendon, QT: quadriceps tendon. Data has been represented as % (N/Annual total).

	2014	2015	2016	2017	2018	2019	2020	2021	2022	R	p	1-β (Power)
QT % of cases	(0/105)	(0/206)	(0/200)	(0/231)	(0/226)	(0/204)	4.2 (6/144)	17.1 (29/170)	24.0 (23/96)	0.985	0.109	0.992
HT % of cases	36.19 (38/105)	36.41 (75/206)	37.5 (75/200)	35.5 (82/231)	42.92 (97/226)	47.06 (96/204)	26.39 (38/144)	14.12 (24/170)	8.33 (8/96)	-0.668	*0.049	0.656
BPTB % of cases	63.81 (67/105)	63.59 (131/206)	62.5 (125/200)	64.5 (149/231)	57.08 (129/226)	52.94 (108/204)	68.06 (98/144)	68.82 (117/170)	67.71 (65/96)	0.268	0.486	0.114

There were no significant differences in QT autograft usage based on surgeon fellowship training, APV, or experience (Table 2). From 2020 to 2022, the annual proportion of autograft reconstructions using QT, and the annual proportion of surgeons who utilized QT for autograft reconstruction, increased each year among nearly all surgeon cohorts (Table 3). This trend was found to be statistically significant among lower-volume surgeons (R=0.999, p<0.01). Conversely, the annual proportion of cases using HT for autograft reconstruction, and the annual proportion of surgeons who utilized HT for autograft reconstruction, decreased each year among nearly all surgeon cohorts. However, this was only found to be significant among sports-trained surgeons (R=-0.998, p=0.037, 1-β>0.99). While many surgeon cohorts trended toward an inverse correlation between QT and HT, none were found to be statistically significant.

**Table 2:** There were no differences in QT autograft usage based on surgeon fellowship training, annual procedure volume, or experience. α, β, z, and p represent z-test of proportions between corresponding cohorts. * Indicates statistical significance (p < 0.05). QT: quadriceps tendon, ACLR: anterior cruciate ligament reconstruction. Data has been represented as N, %, and standard deviation of % in separate columns.

	QT ACLR	Total ACLR	QT Utilization (%)	Standard Deviation (%)	α	z	p	1-β (Power)
Sports-trained	41	1,105	3.7	0.57	0.05	0.142	0.887	0.050
Non-sports-trained	17	477	3.6	0.85				
Higher-volume	23	586	3.9	0.8	0.05	0.412	0.674	0.069
Lower-volume	35	996	3.5	0.58				
High-experience	20	664	3	0.66	0.05	-1.178	0.239	0.216
Low-experience	38	918	4.1	0.66				

**Table 3:** Trends in annual ACLR autograft utilization by surgeon factors (2020-2022). * Indicates statistical significance (p < 0.05). R and p values reflect results of Pearson correlation. HT: hamstring tendon; QT: quadriceps tendon. Data has been represented as % (N/Annual total).

	2020 QT % of Cases	2021 QT % of Cases	2022 QT % of Cases	R	p	1-β (Power)	2020 HT % of Cases	2021 HT % of Cases	2022 HT % of Cases	R	p	1-β (Power)
Sports-trained	3.6 (4/110)	18.4 (23/125)	21.2 (14/66)	0.931	0.238	0.633	22.7 (25/110)	14.4 (18/125)	7.6 (5/66)	-0.998	0.037*	0.999
Non-sports-trained	5.9 (2/34)	13.3 (6/45)	30.0 (9/30)	0.977	0.138	0.956	38.2 (13/34)	13.3 (6/45)	10.0 (3/30)	-0.915	0.265	0.552
Higher-volume	3.7 (2/54)	19.1 (13/68)	20.0 (8/40)	0.89	0.302	0.456	22.7 (15/66)	11.0 (9/82)	7.5 (3/40)	-0.955	0.192	0.791
Lower-volume	4.4 (4/90)	15.7 (16/102)	26.8 (15/56)	0.999	0.003*	0.999	30.0 (27/90)	14.7 (15/102)	8.9 (5/56)	-0.968	0.162	0.892
High-experience	5.2 (3/58)	18.2 (12/66)	21.4 (9/42)	0.944	0.214	0.713	27.6 (16/58)	21.2 (14/66)	4.8 (2/42)	-0.952	0.198	0.769
Low-experience	3.5 (3/86)	16.4 (17/104)	25.9 (14/54)	0.996	0.056	0.999	25.6 (22/86)	9.6 (10/104)	11.1 (6/54)	-0.82	0.387	0.296

## Discussion

The QT has been increasingly utilized in primary autograft ACLR at our large multi-centered institution, with an associated decline in HT autograft usage. Increases in QT autograft utilization were observed across all surgeon cohorts, regardless of fellowship training, APV, or experience. Surgeons who utilized QT autograft were more likely to have a high APV than those who did not utilize the graft. Further, an inverse relationship between QT and HT utilization was demonstrated among non-sports-trained surgeons. Prior analyses of graft utilization in ACLR have reported on the proportion of surgeons who reported QT autograft as their graft of choice through physician surveys; however, literature is limited on studies that analyze graft utilization and have included the QT autograft [22,23,26,27]. To our knowledge, this is the first study to report on annual trends in QT autograft utilization in ACLR at a single institution.

The autograft selection patterns observed can likely be attributed to recent biomechanical and clinical outcome studies that have supported the use of QT autograft for ACLR. The QT autograft offers the highest cross-sectional area of any autograft, and its load to failure exceeds that of the native ACL, with tensile strength reported as high as 2,352 newtons [4-6]. Additionally, it has been shown to have 20% more collagen fibers and a higher density of fibroblasts than BPTB upon histological analysis [4-6]. Although the HT tensile strength outperforms the QT in some biomechanical studies, recent studies found no difference between QT and HT in knee function with regards to load bearing and kinematics [3,5,17].

Recent literature has indicated that QT autograft ACLR performs similar or superior to HT in donor-site morbidity and graft failure rates [6,18,20]. QT autograft ACLR has been shown to result in favorable subjective and objective metrics, including Lysholm score, knee laxity, and knee stability [6,18,20,21]. Clinically, the quadriceps muscle recovery after QT ACLR has been found to be equivalent to that of BPTB and HT autograft, alleviating previous concerns regarding the impact of QT harvest on extensor mechanism function [6,28]. One recent study found that harvesting hamstring tendons can significantly reduce the knee's muscular protection during side-step cutting two years after ACLR, contributing to ipsilateral reinjury [29]. While many studies have highlighted the drawbacks of the HT autograft, an unreliable graft diameter is most notable, as a small diameter can increase the risk of graft failure [11,12]. In contrast, the QT autograft produces a more predictable graft size [4-6]. Further, utilization of the QT autograft provides the surgeon with the option of harvesting a bone block from the superior pole of the patella, which can offer advantages similar to those provided by the BPTB autograft, such as bone to bone healing [17].

A recent biannual survey study of the ACL Study Group surgeons observed changes in ACLR autograft preference over time and found that there was an increasing proportion of surgeons who preferred QT autograft from 2014 to 2018. This same study demonstrated a peak in HT preference in 2014, with a consistently decreased proportion of surgeons who preferred HT in subsequent years [22]. Geographical patterns and preferences likely influence graft selection in ACLR, and thus, QT utilization may vary between different regions. A global survey on ACLR preferred graft choice distributed in 2020 found that only 2.4% of the 2,107 respondents favored QT autografts, and North American surgeons were less likely to list the HT autograft as their preferred graft choice than surgeons of other continents [23].

Fellowship training has been shown to influence the technical preferences of surgeons in other sports medicine procedures and likely impacts operative decisions in ACLR [30]. In autograft ACLR cases from 2020 to 2022, QT utilization increased with an associated decrease in HT utilization across nearly all surgeon cohorts, but a significant inverse correlation between these two rates was only observed among high-volume surgeons (R=0.998, p=0.035). This suggests that high-volume surgeons may have begun to use QT in cases that they would have previously utilized HT autograft. Additionally, from 2020 to 2022, sports-trained surgeons were the only surgeon cohort which demonstrated a significant decrease in HT autograft utilization, indicating a particularly strong movement away from HT by these surgeons (R=-0.998, p=0.037).

Other surgeon characteristics accounted for by this study included case volume and years of experience. Lower-volume ACLR surgeons were the only subcategory of surgeons that demonstrated a significant increase in QT utilization after initial adoption at the study institution in 2020. This group may have been more likely to experience complications in ACLR [25], which could have resulted in these surgeons seeking out an alternative autograft choice more frequently. Only low-experience surgeons demonstrated significant increases in QT autograft utilization between 2020 and 2022, which could be attributed to less established preferences of these surgeons, who may be more receptive to new research.

Limitations

As this analysis was limited to a single institution, the findings may reflect institutional or regional trends in autograft selection rather than broadly generalizable patterns. The retrospective design and lack of follow-up clinical outcome data also precluded assessment of patient-reported outcomes and limited the ability to interpret the observed trends.

The QT autograft was not adopted into clinical practice at our institution until 2020, resulting in a smaller sample size for QT ACLR compared to other graft types. This may limit the reproducibility of observed trends, particularly in subgroup analyses. Notably, no singular event or policy change was identified to explain the marked increase in QT utilization after 2020, and no survey data were collected to clarify the basis of this shift. Some statistically significant trends, especially among smaller surgeon subgroups, may reflect sampling variability rather than true divergence in clinical practice.

The dichotomy of 15 years in clinical practice was chosen arbitrarily, limiting interpretation of results related to surgeon experience. Additionally, the study focused solely on autograft ACLR procedures; allograft utilization was not evaluated. Although multiple statistical tests were performed, corrections for multiple comparisons were not applied. This may modestly increase the likelihood of type I error; however, given the exploratory and descriptive nature of the analysis, these results should be interpreted cautiously and confirmed through future research.

## Conclusions

The increasing utilization of QT autograft for ACLR reflects a shift in surgical preference at the study institution since 2020, corresponding with a decline in HT autograft use. This trend was observed consistently across surgeon cohorts, including both sports and non-sports fellowship-trained surgeons, high- and low-experience surgeons, and those with higher and lower annual procedure volumes. The shift may be driven by emerging biomechanical and clinical evidence supporting the QT autograft’s advantages, such as predictable graft size, high tensile strength, and favorable clinical outcomes. While HT autograft utilization has declined, particularly among sports-trained surgeons, regional and institutional factors may still influence graft selection patterns.

Future research should focus on multicenter analyses to confirm these findings and assess long-term patient outcomes associated with QT autograft ACLR, including knee laxity, donor-site pain, quadriceps strength, and revision rate. Additionally, further investigation into the impact of surgeon training, experience, and case volume on graft selection may provide insights into the adoption of new techniques. Despite the study’s limitations, our findings demonstrate an increased preference for QT autograft at the study institution and indicate that it may become a more prominent option in ACLR moving forward.

## References

[1] Zaffagnini S, Grassi A, Serra M, Marcacci M (2015). Return to sport after ACL reconstruction: how, when and why? A narrative review of current evidence. Joints.

[2] MARS Group (2016). Factors influencing graft choice in revision anterior cruciate ligament reconstruction in the MARS group. J Knee Surg.

[3] Lin KM, Boyle C, Marom N, Marx RG (2020). Graft selection in anterior cruciate ligament reconstruction. Sports Med Arthrosc Rev.

[4] Shani RH, Umpierez E, Nasert M, Hiza EA, Xerogeanes J (2016). Biomechanical comparison of quadriceps and patellar tendon grafts in anterior cruciate ligament reconstruction. Arthroscopy.

[5] Runer A, Keeling L, Wagala N, Nugraha H, Özbek EA, Hughes JD, Musahl V (2023). Current trends in graft choice for anterior cruciate ligament reconstruction: part I: anatomy, biomechanics, graft incorporation and fixation. J Exp Orthop.

[6] Mouarbes D, Menetrey J, Marot V, Courtot L, Berard E, Cavaignac E (2019). Anterior cruciate ligament reconstruction: a systematic review and meta-analysis of outcomes for quadriceps tendon autograft versus bone-patellar tendon-bone and hamstring-tendon autografts. Am J Sports Med.

[7] Dai W, Leng X, Wang J, Cheng J, Hu X, Ao Y (2022). Quadriceps tendon autograft versus bone-patellar tendon-bone and hamstring tendon autografts for anterior cruciate ligament reconstruction: a systematic review and meta-analysis. Am J Sports Med.

[8] Xie X, Liu X, Chen Z, Yu Y, Peng S, Li Q (2015). A meta-analysis of bone-patellar tendon-bone autograft versus four-strand hamstring tendon autograft for anterior cruciate ligament reconstruction. Knee.

[9] Marques FD, Barbosa PH, Alves PR (2020). Anterior knee pain after anterior cruciate ligament reconstruction. Orthop J Sports Med.

[10] Kartus J, Movin T, Karlsson J (2001). Donor-site morbidity and anterior knee problems after anterior cruciate ligament reconstruction using autografts. Arthroscopy.

[11] Magnussen RA, Lawrence JT, West RL, Toth AP, Taylor DC, Garrett WE (2012). Graft size and patient age are predictors of early revision after anterior cruciate ligament reconstruction with hamstring autograft. Arthroscopy.

[12] Vardiabasis N, Mosier B, Walters J, Burgess A, Altman G, Akhavan S (2019). Can we accurately predict the quadruple hamstring graft diameter from preoperative magnetic resonance imaging?. Orthop J Sports Med.

[13] Barker JU, Drakos MC, Maak TG, Warren RF, Williams RJ 3rd, Allen AA (2010). Effect of graft selection on the incidence of postoperative infection in anterior cruciate ligament reconstruction. Am J Sports Med.

[14] Samuelsson K, Andersson D, Karlsson J (2009). Treatment of anterior cruciate ligament injuries with special reference to graft type and surgical technique: an assessment of randomized controlled trials. Arthroscopy.

[15] Cristiani R, Sarakatsianos V, Engström B, Samuelsson K, Forssblad M, Stålman A (2019). Increased knee laxity with hamstring tendon autograft compared to patellar tendon autograft: a cohort study of 5462 patients with primary anterior cruciate ligament reconstruction. Knee Surg Sports Traumatol Arthrosc.

[16] Fulkerson JP, Langeland R (1995). An alternative cruciate reconstruction graft: the central quadriceps tendon. Arthroscopy.

[17] Sasaki N, Farraro KF, Kim KE, Woo SL (2014). Biomechanical evaluation of the quadriceps tendon autograft for anterior cruciate ligament reconstruction: a cadaveric study. Am J Sports Med.

[18] Slone HS, Romine SE, Premkumar A, Xerogeanes JW (2015). Quadriceps tendon autograft for anterior cruciate ligament reconstruction: a comprehensive review of current literature and systematic review of clinical results. Arthroscopy.

[19] Lund B, Nielsen T, Faunø P, Christiansen SE, Lind M (2014). Is quadriceps tendon a better graft choice than patellar tendon? A prospective randomized study. Arthroscopy.

[20] Cavaignac E, Coulin B, Tscholl P, Nik Mohd Fatmy N, Duthon V, Menetrey J (2017). Is quadriceps tendon autograft a better choice than hamstring autograft for anterior cruciate ligament reconstruction? A comparative study with a mean follow-up of 3.6 years. Am J Sports Med.

[21] Nyland J, Collis P, Huffstutler A, Sachdeva S, Spears JR, Greene J, Caborn DN (2020). Quadriceps tendon autograft ACL reconstruction has less pivot shift laxity and lower failure rates than hamstring tendon autografts. Knee Surg Sports Traumatol Arthrosc.

[22] Arnold MP, Calcei JG, Vogel N (2021). ACL Study Group survey reveals the evolution of anterior cruciate ligament reconstruction graft choice over the past three decades. Knee Surg Sports Traumatol Arthrosc.

[23] Tuca M, Valderrama I, Eriksson K, Tapasvi S (2023). Current trends in anterior cruciate ligament surgery: a worldwide benchmark study. J ISAKOS.

[24] Middleton KK, Hamilton T, Irrgang JJ, Karlsson J, Harner CD, Fu FH (2014). Anatomic anterior cruciate ligament (ACL) reconstruction: a global perspective: part 1. Knee Surg Sports Traumatol Arthrosc.

[25] Schairer WW, Marx RG, Dempsey B, Ge Y, Lyman S (2017). The relation between volume of ACL reconstruction and future knee surgery. Orthop J Sports Med.

[26] van Eck CF, Schreiber VM, Mejia HA, Samuelsson K, van Dijk CN, Karlsson J, Fu FH (2010). "Anatomic" anterior cruciate ligament reconstruction: a systematic review of surgical techniques and reporting of surgical data. Arthroscopy.

[27] Kaeding CC, Pedroza AD, Reinke EK, Huston LJ, Hewett TE, Flanigan DC, Spindler KP (2017). Change in anterior cruciate ligament graft choice and outcomes over time. Arthroscopy.

[28] Iriuchishima T, Ryu K, Okano T, Suruga M, Aizawa S, Fu FH (2017). The evaluation of muscle recovery after anatomical single-bundle ACL reconstruction using a quadriceps autograft. Knee Surg Sports Traumatol Arthrosc.

[29] Konrath JM, Killen BA, Saxby DJ (2023). Hamstring harvest results in significantly reduced knee muscular protection during side-step cutting two years after anterior cruciate ligament reconstruction. PLoS One.

[30] Kelly BC, Constantinescu DS, Pavlis W, Vap AR (2021). Arthroscopic versus open rotator cuff repair: fellowship-trained orthopaedic surgeons prefer arthroscopy and self-report a lower complication rate. Arthrosc Sports Med Rehabil.

